# Gut microbiota and targeted metabolomics of short-chain fatty acid reveals Qing-Re-Zao-Shi-Jian-Pi prescription on glucose and lipid metabolism in type 2 diabetic mice

**DOI:** 10.3389/fnut.2025.1626778

**Published:** 2025-09-30

**Authors:** Jing Liu, Meng Qin, Haidan Wang, Yingzhuo Ran, Xin Shao

**Affiliations:** 1 Department of Endocrinology, Nanjing Hospital of Chinese Medicine Affiliated to Nanjing University of Chinese Medicine, Nanjing, Jiangsu, China; 2 State Key Laboratory of Natural Medicines, School of Traditional Chinese Pharmacy, China Pharmaceutical University, Nanjing, Jiangsu, China; 3 Jiangsu Province Hospital of Chinese Medicine, Affiliated Hospital of Nanjing University of Chinese Medicine, Nanjing, Jiangsu, China

**Keywords:** type 2 diabetes, microbiota, short-chain fatty acid, Chinese medicine, insulin resistance

## Abstract

**Background:**

Type 2 diabetes (T2D) is a group of metabolic disorders characterized by chronic hyperglycemia which caused by insufficient insulin secretion or defective insulin action. It has been found that the Qing-Re-Zao-Shi-Jian-Pi prescription (QRZSF) can effectively treat T2D in clinic, but the mechanism of action is unclear.

**Methods:**

The T2D mouse model was constructed using combination of high-fat diet and intraperitoneal injection of Streptozocin. The levels of blood glucose, lipids, insulin resistance (IR), Interleukin-6 (IL-6), interleukin-1β (IL-1β), tumor necrosis factor-α (TNF-α) were detected, and the pancreatic tissue damage was evaluated by Hematoxylin and eosin (H&E) staining microscope. 16S rDNA high-throughput sequencing was performed to analyze the intestinal flora and LC-MS was used to detect the contents of short-chain fatty acids (SCFAs).

**Results:**

The fasting blood glucose, blood glucose during oral glucose tolerance test (OGTT), insulin, Homeostatic Model Assessment of Insulin Resistance (HOMA-IR), Triglyceride (TG), low-density lipoprotein cholesterol (LDL-C), Total cholesterol (TCHO), IL-1β, TNF-α were significantly decreased in QRZSF group compared with the model group. The damage of pancreatic tissue was improved, and the butyrate level was significantly increased. Besides, QRZSF group had a significant effect on intestinal flora profiling in T2D mice. Compared with the model group at the phylum level, the abundance of *Proteobacteria*, *Chloroflexi*, *Fusobacteriota*, *Cyanobacteria* were increased, while the abundance of *Patescibacteria* and *Verrucomicrobiota* were decreased in QRZSF group. At the genus level, the abundance of *Anaerotignum, Akkermansia, Candidatus_ Saccharimonas* were decreased, while the abundance of *Acetatifactor* and *Bacteroides* were increased. The possible mechanism is related to adjusting the abundance of gut microbiota and increasing *Bacteroides* and butyrate to reduce inflammation.

**Conclusion:**

Our research showed that the QRZSF can correct the disorder of glucose and lipid metabolism, reduce the level of proinflammatory factors, improve IR and pancreatic tissue damage, regulate the diversity of gut microbiota, increase the content of butyrate in SCFAs, and then effectively prevent and delay the occurrence and development of diabetes. The study provides a new idea for the prevention and treatment of T2D with traditional Chinese medicine.

## 1 Background

Type 2 diabetes (T2D) is a complex, genetic and heterogeneous disorder, clinically manifesting as chronic hyperglycemia. This pathophysiological condition emerges from progressive β-cell dysfunction, frequently compounded by insulin resistance (IR) (1, 2). As society evolve and daily habits shift, these combined factors are driving up rates of T2D around the world, and its affected population tends to be younger. The multisystem complications associated with T2D pose significant threats to patient health while imposing substantial economic burdens on affected individuals and healthcare systems. Recognized as a complex chronic metabolic disorder, T2D has emerged as a critical public health challenge requiring urgent intervention. The incidence of this disease has family aggregation and genetic tendency. T2D typically presents with a gradual and subtle onset, frequently remaining asymptomatic during initial disease progression. It is the reason why this disease can often not find and diagnosed in time. With the development of T2D, patients will have arteriosclerosis and microangiopathy, which will cause many chronic complications of diabetes, such as diabetic macroangiopathy, diabetic retinopathy, diabetic nephropathy, and diabetic peripheral neuropathy. Long-term complications from diabetes often lead to severe disability and higher death rates in patients. Because of this, starting treatment early for T2D can greatly improve patients’ future health outcomes. At present, commonly used drugs for treating T2D include oral hypoglycemic agents, insulin and glucagon-like peptide -1 receptor agonist. Among them, oral hypoglycemic drugs are divided into biguanides, sulfonylureas, glinides, α-glucosidase inhibitors, thiazolidinediones, dipeptidyl peptidase IV (DPP-4) inhibitors and sodium-glucose cotransporter 2 (SGLT2) inhibitors due to different mechanisms of action. T2D is a progressive disease, and the curative effect of most patients decreases after taking a single oral medicine for a period. After that it needs to be treated with multiple drugs. All kinds of hypoglycemic drugs have different side effects. Using multiple drugs together raises both health risks and treatment costs for patients. The important mechanism of T2D is IR related to chronic inflammation, and IR runs through the whole course of T2D. Recent research indicates that shifts in intestinal flora influence IR progression, which mainly plays a role by intervening in inflammatory pathways (3, 4). Intestinal flora imbalance affects insulin signal transduction and gene expression of islet β cells by inducing inflammatory reaction and expression and secretion of cytokines, thus causing IR. Intestinal flora is being regarded as a potential therapeutic strategy for T2D.

Qing-Re-Zao-Shi-Jian-Pi prescription (QRZSF) based on this treatment evolved from the traditional famous prescription ErMiao San. ErMiao San is composed of *Phellodendri Chinensis Cortex* and *Atractylodis Rhizoma*. Under the experience of clinical application, we optimized the ancient prescription. The QRZSF is composed of five kinds of Chinese herbal medicine, namely *Atractylodis Rhizoma*, *Phellodendri Chinensis Cortex*, *Scutellariae Radix*, *Coptidis Rhizoma* and *Coicis Semen*. The prescription has curative effects on diabetes, but the mechanism is not clear.

Our preliminary clinical research found this prescription had clear effects on T2D and IR with patients (5, 6), and the intestinal flora of T2D patients had differential expression (7). Therefore, this study explored the mechanism of QRZSF in the intervention of IR in T2D from the perspective of intestinal flora. We established the T2D mouse model using high-fat feeding and low-dose streptozotocin injections. These animals then received daily treatments with the QRZSF prescription. After 4 weeks of treatment, the QRZSF group showed marked decreases in fasting blood glucose and post-meal glucose levels compared to model mice. Insulin levels and IR scores (HOMA-IR) were also reduced. Additionally, pancreatic islet lesions showed clear improvement. The pancreatic islet cell degeneration score also dropped noticeably. Regarding blood lipid levels, the QRZSF group exhibited a marked reduction in Triglyceride (TG), low-density lipoprotein cholesterol (LDL-C), and Total cholesterol (TCHO) when compared to the model group. Furthermore, the concentrations of inflammatory markers, specifically interleukin-1β (IL-1β) and tumor necrosis factor-α (TNF-α), were observed to decline significantly in the QRZSF group relative to the model group. The content of short-chain fatty acids (SCFAs) in feces was determined using LC-MS detection method for T2D mice. Results revealed that butyrate was present in notably greater amounts in the QRZSF group than in the model group while propionate and acetate contents did not show significant differences. The study employed 16S rDNA high-throughput sequencing to assess the composition of the intestinal flora and found that at the phylum level, the *Proteobacteria*, *Chloroflexi*, *Fusobacteriota*, and *Cyanobacteria* abundances were increased, while the QRZSF group exhibited reduced abundances of *Patescibacteria* and *Verrucomicrobiota* relative to the model group. At the level of bacterial group categorization by genus, the abundance of *Anaerotignum*, *Akkermansia*, *Candidatus_ Saccharimonas* were decreased, while the QRZSF group displayed elevated levels of *Acetatifactor* and *Bacteroides* compared to the model group. We found the possible mechanism for reducing the level of glucose, lipid metabolism and IR in T2D, which is linked to regulating the abundance of intestinal flora, increasing bacteroides and reducing inflammation with butyrate.

## 2 Materials and methods

### 2.1 Animals and drugs

Fifty healthy male C57BL/6 mice (8 weeks-old, SPF-grade, 18–22 g body weight) were obtained from Changzhou Cavens Laboratory Animal Co., Ltd [SCXK (Su) 2021-0013 licensed provider]. This study received official approval from China Pharmaceutical University’s Animal Ethics Committee, which strictly adhered to the ethical and welfare guidelines for experimental animals to minimize the suffering of animals during animal experiments. Traditional Chinese medicine prescriptions for QRZSF consists of *Atractylodis Rhizoma* (15 g), *Phellodendri Chinensis Cortex* (6 g), *Scutellariae Radix* (3 g), *Coptidis Rhizoma* (10 g), and *Coicis Semen* (30 g). These Chinese medicines are purchased from the traditional Chinese medicine pharmacy of Nanjing traditional Chinese Medicine Hospital Affiliated to Nanjing University of traditional Chinese medicine. Metformin hydrochloride tablets (North China Pharmaceutical Co., Ltd., gyzz h20113492, specification: 0.5 g/tablet) were purchased from the Western pharmacy of Nanjing traditional Chinese Medicine Hospital Affiliated to Nanjing University of traditional Chinese medicine.

### 2.2 Animal modeling and grouping

All mice were kept in room (temperature 22 ± 1 °C, 12 h light dark cycle, 45%–65% relative humidity) with continuous access to food and water throughout the study period. After a 7 days period with standard diet to ensure environmental adaptation, all mice were measured for weight and fasting blood glucose, and their feces were collected. When the samples were collected completely, 10 mice in the control group were randomly selected to be fed with ordinary full price nutritional feed, and the remaining 40 mice assigned to the model group received a customized formulated diet rich in sugars and fats. After 4 weeks of feeding with special feed, the model group was intraperitoneally injected with 2% STZ solution at the dose of 100 mg/kg, and the fasting blood glucose levels were checked through tail vein blood samples after 1 week. Mice with fasting blood glucose value ≥ 11.1 mmol/l were included in the experiment to establish model successfully. The T2D model mice were assigned to four distinct groups (10 mice per group) using a randomization protocol: model group, metformin group (0.2 g/kg/day), low-dose (QRZSF-L) group (5 g/kg/day), high-dose (QRZSF-H) group (10 g/kg/day). The last three groups received daily medication treatments tailored to their group designation for four consecutive weeks. Control and model groups were provided with equal volumes of distilled water following the same administration schedule.

### 2.3 Oral glucose tolerance test (OGTT) and insulin tolerance test (ITT)

Oral glucose tolerance test experiments were conducted, with each mouse given 20% glucose solution at a rate of 2 g/kg by gavage. Blood glucose levels were determined at the tail vein at 0, 30, 60, and 120 min, respectively. Fasting serum insulin and C-peptide levels were detected according to the kit instructions (Nanjing Boyan Biotechnology Co., Ltd. Nanjing, China). Calculate IR index: HOMA-IR = FBG (mmol/L) * FINS (mIU/L)/22.5.

### 2.4 Biochemical assays

Total cholesterol, TG, high-density lipoprotein cholesterol (HDL-C) and LDL-C assay kits were provided by Nanjing Boyan Biotechnology Co., Ltd. (Nanjing, China).

### 2.5 ELISA

ELISA was used to quantify the serum concentrations of inflammatory factors in mice: TNF- α, Interleukin-6 (IL-6), IL-1β.

### 2.6 Hematoxylin and eosin (H&E) staining

At the end of the experiment, pancreatic tissue was immersion-fixed in 10% formaldehyde solution, routinely taken, dehydrated, embedded in paraffin, sliced (4 μm thick) and stained with hematoxylin-eosin. H&E-stained pancreatic sections underwent microscopic examinations to evaluate tissue abnormalities. Pathological scores ranged from 0 to 4, directly reflecting observed tissue damage severity in histological evaluations, where “0” was no obvious change and “4” was serious pathological change.

### 2.7 Detection of short chain fatty acids

#### 2.7.1 Sample preparation

A total of 1 ml acetonitrile was added to 10 mg of fecal (intestinal contents) homogenate, centrifuge at 13,000 rpm for 10 min, and 40 μL supernatant was taken. The supernatant was added with 5 μL d3-hexanoic acid (10 μg/mL), vortex for 3 min, then add 20 μL of 3 NPH (200 mM) and 20 μL of EDC-HCl-6% pyridine (120 mM) solution. After vortexing for 3 min, and then react at 40 °C for 30 min. The samples were then centrifuged at 18,000 rpm for 10 min and the supernatant analyzed.

#### 2.7.2 Preparation of standard curve

A total of 10 μL acetic acid (1.32 mg/ml), 10 μL propionic acid (1.03 mg/ml), 1 μL butyric acid (1.00 mg/ml) and 67.9 μL methanol were added into 1.5 ml tubes, respectively. The mixed solution of acetic acid (132 ng/ml), propionic acid (103 ng/ml) and butyric acid (10 ng/ml) was obtained. The standard solution of each concentration gradient is prepared by half-diluting the mixed solution with methanol. The mixed solution of each concentration of 40 μL was taken and treated with the sample in the same step.

#### 2.7.3 LC-MS analysis

The samples were analyzed using an UltiMate^®^ 3000 ultra-performance liquid chromatography system (DIONEX, Sunnyvale, CA, United States) coupled with an Acquity UPLC^®^ BEH C18 column (2.1 × 100 mm, 1.7 μm, Waters Co., Milford, MA, United States). Separation was achieved on BDS HYPERSIL-C18 (100 mm × 2.1 mm, 2.4 μm). Mobile phase A was aqueous solution containing 0.1% formic acid (*v*/*v*), while mobile phase B was 0.1% formic acid acetonitrile (*v*/*v*). The mobile phase gradient was as follows: 0–3 min, 10% B, 3–10 min, 10%–35% B, 10–12 min, 35%–95% B, 12–14 min, 95% B, 14–14.5 min, 95%–10% B. The flow rate was 0.3 mL/min. The ion source was an electrospray ion source (ESI ion source), and the scanning mode of multiple reaction monitoring (MRM) was monitored in negative ion mode, with a spray voltage of 3,200 V, a sheath gas pressure of 45 bar, an auxiliary gas pressure of 25 bar, a gasification tube temperature of 300 °C, a capillary temperature of 350 °C.

#### 2.7.4 Date analysis

Processing of the LC-MS raw data was conducted through MassLynx software (version 4.1, developed by Waters, located in Milford, MA, United States). This involved integrating peaks, calibrating, and quantifying each metabolite. Subsequently, the statistical analysis was executed with Prism 8 (from GraphPad) and R (version 3.5.1) (8).

### 2.8 16 S rDNA gene amplification and sequencing

At the final stage of the experiment, total DNA was extracted from mouse fecal samples in all groups using the CTAB method (cetyltrimethylammonium bromide). To evaluate the extracted DNA quality, agarose gel electrophoresis was performed. DNA concentration was then measured with a UV spectrophotometer. For bacterial community analysis, PCR amplification targeted the V3–V4 hypervariable regions of the 16S rDNA gene. The primer pair 341F (5′-CCTACGGGNGGCWGCAG-3′) and 805R (5′-GACTACHVGGGTATCTAATCC-3′) was used in this process. After amplification, PCR products were first purified with Ampure XT beads and quantified using a Qubit fluorometer. Based on the pre-calculated sequencing requirements, purified products from individual samples were pooled at appropriate ratios. To confirm amplification success, a 2% agarose gel electrophoresis step was conducted. The Ampure XT bead-based purification kit was applied for fragment recovery. Purified DNA libraries underwent two quality checks: (1) size distribution analysis using the Agilent 2100 Bioanalyzer, and (2) concentration measurement with the Illumina Library Quantification Kit. For sequencing preparation, qualified libraries were diluted in a gradient series. These diluted samples were then combined according to the required sequencing depth. Finally, single-stranded DNA templates were generated by NaOH denaturation prior to online sequencing. Based on the OTU, Alpha and Beta diversity analyses were performed. Calculate Alpha diversity based on Chao1 index. The between-sample diversity was visualized by a principal coordinate analysis (PCoA) plot. The RDP classifier software was applied to classify the OTU representative sequences, yielding species annotation information at the phylum and genus levels, and facilitating statistical analysis of community structure. The relative abundance of bacterial classification, the alpha and beta diversity of QIIME2 process analysis, and using R (v4.1.0) map drawing.

### 2.9 Statistical analysis

We analyzed the data with SPSS 20.0 software. Numerical results are presented as mean ± standard deviation ($\bar{\text{X}}$ ± s), and the data conforming to the normal distribution were subject to the homogeneity test of variance, the one-way ANOVA was conducted for the comparison between multiple groups, and the Welch test was conducted for the non-homogeneous variance. For groups meeting the homogeneity of variance requirement, we applied the LSD method for intergroup comparisons. We used the Kruskal-Wallis H test for non-normally distributed data. *P* < 0.05 was statistically significant.

## 3 Results

### 3.1 Anti-diabetic effects of QRZSF

**FIGURE 1:**
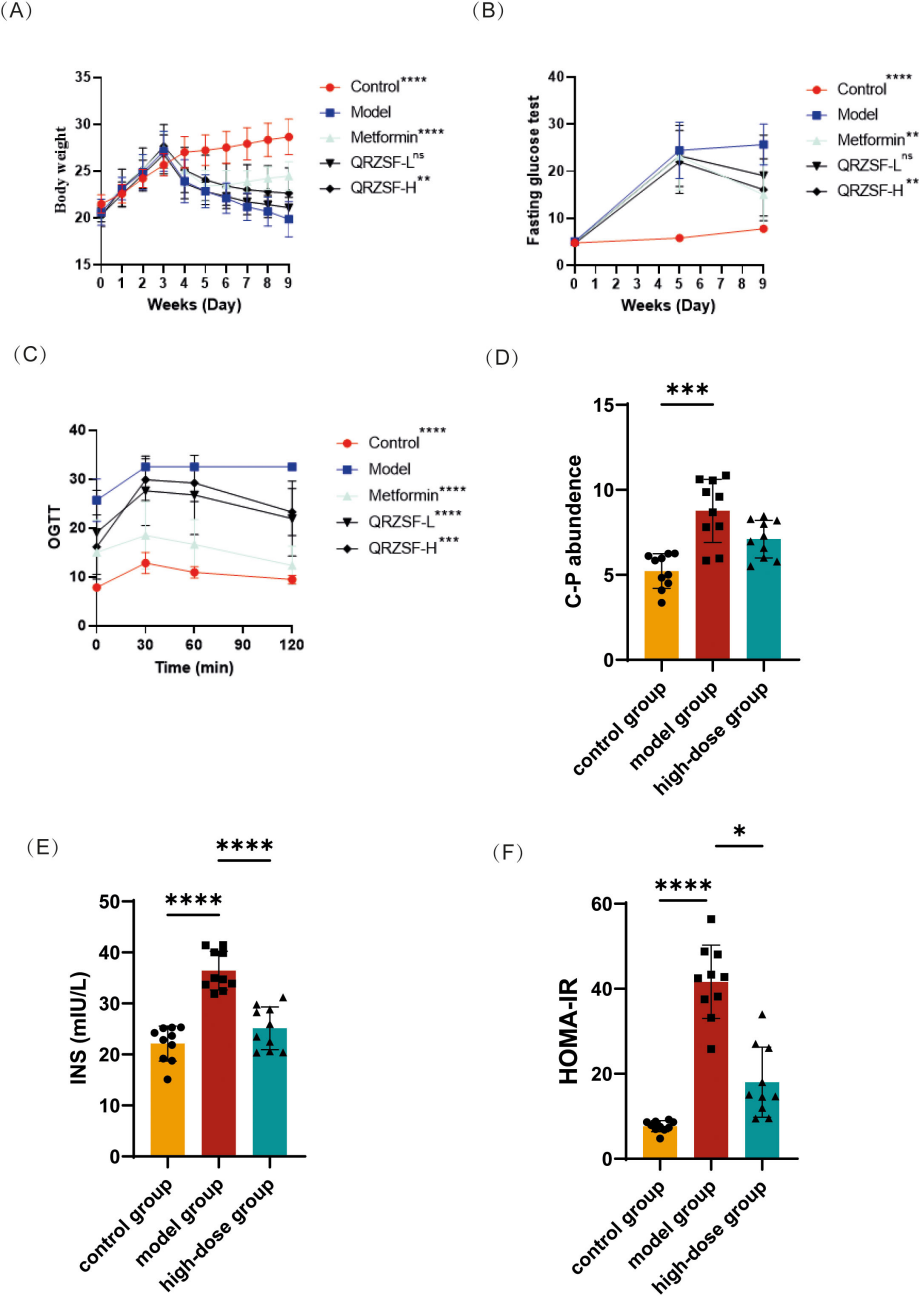
Evaluation of Qing-Re-Zao-Shi-Jian-Pi prescription (QRZSF) on glycometabolism and pancreatic function in the type 2 diabetes (T2D) mouse model using body weight, fasting blood glucose, oral glucose tolerance test (OGTT), C-peptide, insulin and Homeostatic Model Assessment for Insulin Resistance (HOMA-IR). **(A)** D-Effect of QRZSF on body weight in mice; **(B)** Effect of QRZSF on fasting blood glucose; **(C)** E-Effect of QRZSF on OGTT; **(D)** Effect of QRZSF on C-peptide; **(E)** Effect of QRZSF on insulin; **(F)** Effect of QRZSF on HOMA-IR. *P < 0.05, **P < 0.01, ***P < 0.001, ****P < 0.0001.

The body weight of mice after treatment showed an overall downward trend in model, metformin and QRZSF groups compared with the control group (Figure 1A). Compared to the model group, mice in the metformin and QRZSF-H groups showed a substantial increase in body weight after treatment, while the QRZSF-L group had no significant effect. The model group exhibited significantly higher fasting blood glucose levels than the control group. Following 4 weeks of treatment, both metformin and QRZSF-H groups demonstrated significantly lower fasting blood glucose levels compared to the model group, whereas the QRZSF-L group showed no significant reduction (Figure 1B). In OGTT study, the blood glucose of mice in each group increased after glucose gavage and peaked at 30 min. The blood glucose at the end of OGTT was decreased significantly in the control, metformin, QRZSF-H, and QRZSF-L groups compared with model group (Figure 1C).

According to the above research results, it has been found that the QRZSF-H group had better hypoglycemic effect on T2D mice. Therefore, this study chose the high-dose group for further analysis. Compared to controls, the model group showed significantly elevated levels of C-peptide, insulin, and HOMA-IR (Figures 1D–F). Treatment with QRZSF-H group significantly reduced insulin levels and HOMA-IR versus the model group, though it had no significant effect on C-peptide levels.

### 3.2 Effects of QRZSF on blood lipids and inflammatory factors

**FIGURE 2:**
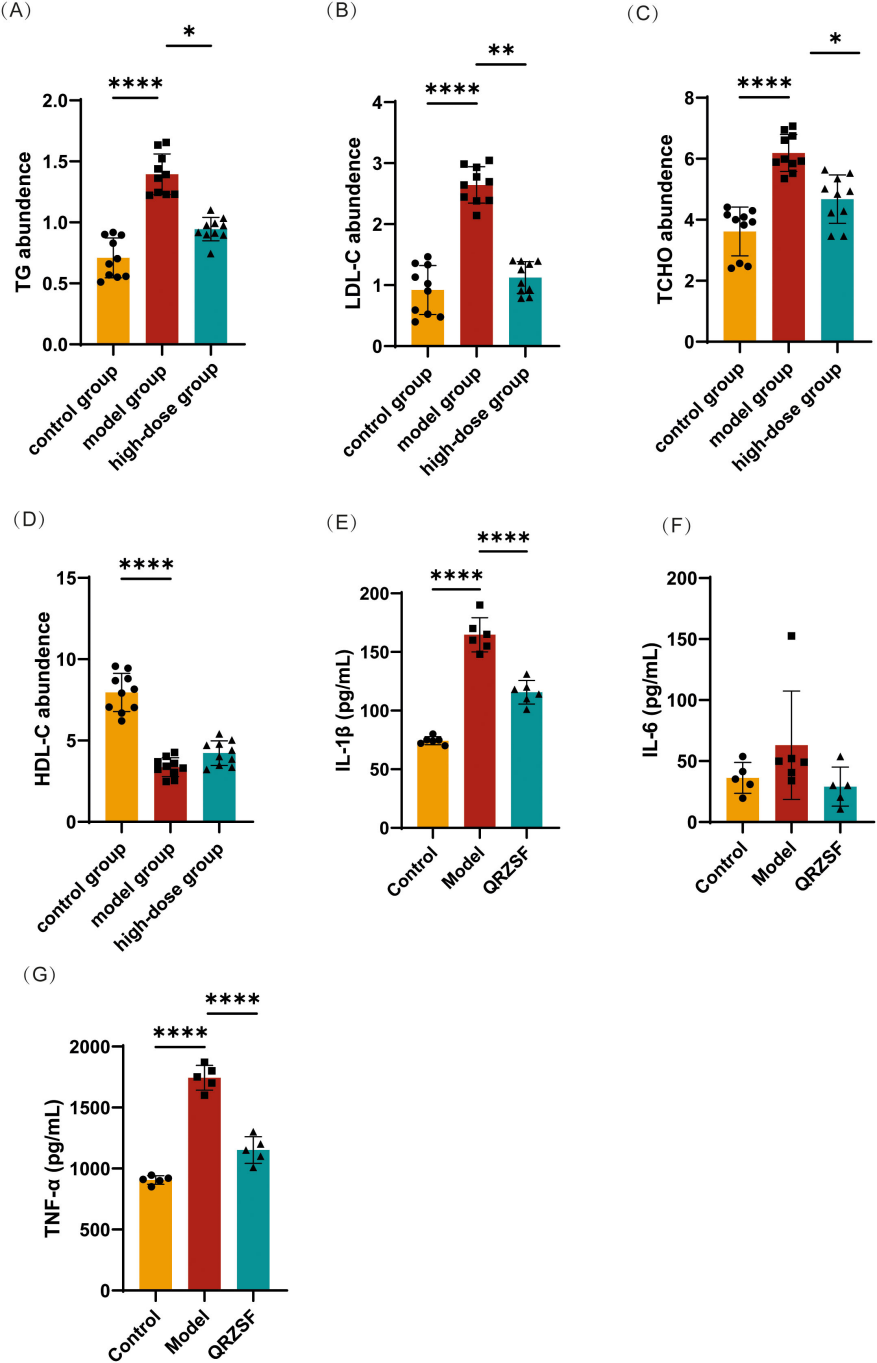
Effect of Qing-Re-Zao-Shi-Jian-Pi prescription (QRZSF) on lipid metabolism and inflammatory factors. **(A–D)** Changes of Triglyceride (TG), low-density lipoprotein cholesterol (LDL-C), total cholesterol (TCHO), and high-density lipoprotein cholesterol (HDL-C) in serum after administration of QRZSF; **(E–G)** Changes of interleukin-1β (IL-1β), interleukin-6 (IL-6), and tumor necrosis factor-α (TNF-α) in serum after administration of QRZSF. *P < 0.05, **P < 0.01, ****P < 0.0001.

The model group showed significantly elevated TG, LDL-C, and TCHO levels along with reduced HDL-C levels versus controls (Figures 2A–D). QRZSF-H treatment group significantly decreased TG, LDL-C, and TCHO levels compared to the model group, but showed no significant effect on HDL-C.

While the model group demonstrated significantly elevated IL-1β and TNF-α levels compared to controls (Figures 2E, G), QRZSF-H treatment group produced a marked reduction in both proinflammatory cytokines (Figures 2E, G). There was no significant difference in IL-6 among the control group, model and QRZSF-H group (Figure 2F).

### 3.3 Effects of QRZSF on short chain fatty acid

**FIGURE 3:**
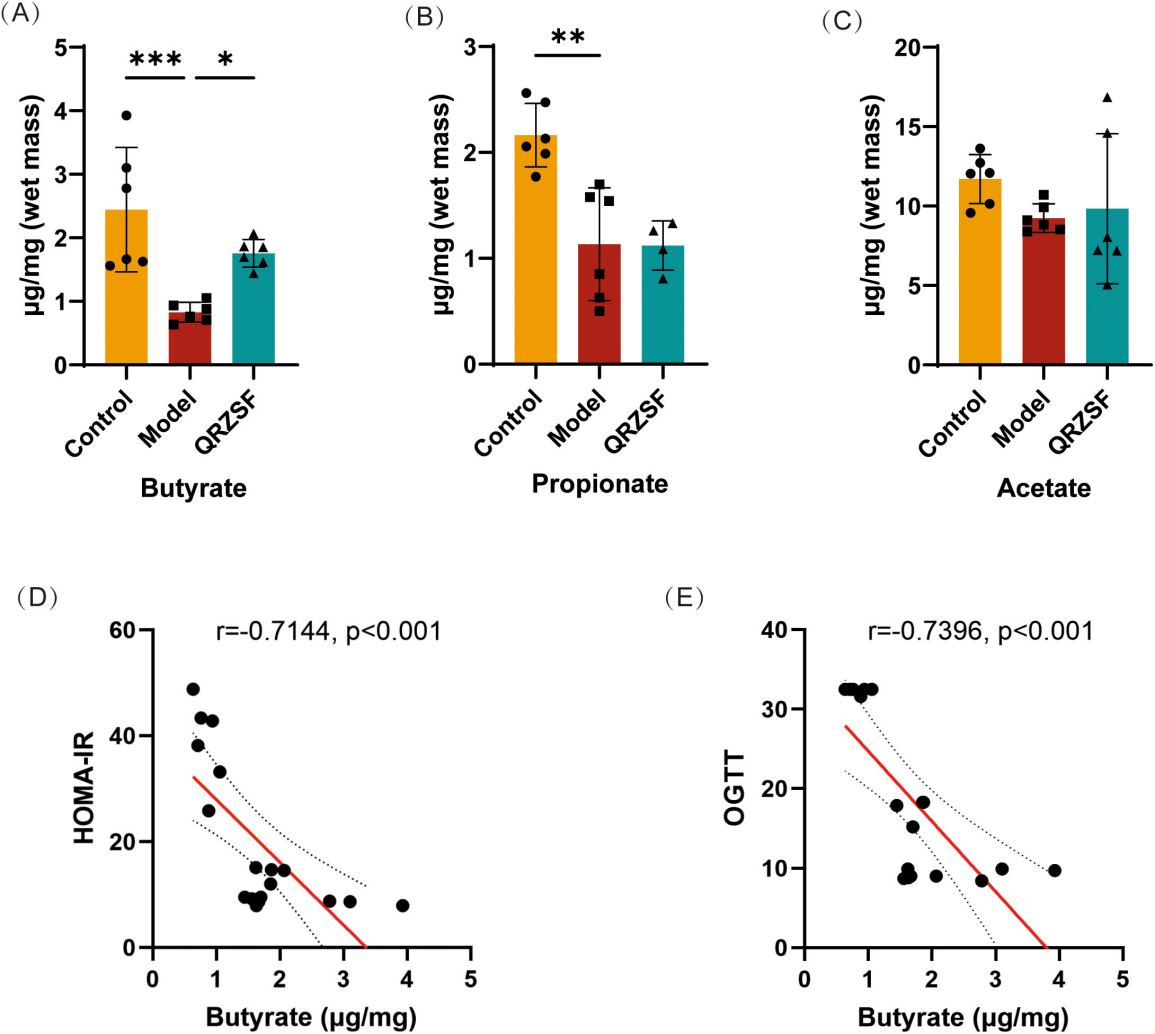
Effect of Qing-Re-Zao-Shi-Jian-Pi prescription (QRZSF) on short chain fatty acid content in intestinal contents. **(A–C)** Changes of butyrate, propionate and acetate after administration of QRZSF; **(D,E)** Pearson correlation analysis between butyrate and phenotype. *P < 0.05, **P < 0.01, ***P < 0.001.

The level of butyrate and propionate were significantly decreased in model group compared with the control group (Figures 3A, B). The level of butyrate was significantly increased in QRZSF-H group compared with model group, while the level of propionate was no-significant effect (Figures 3A, B). There was no significant difference in the level of acetate among the control, model and QRZSF-H group (Figure 3C). Pearson correlation analysis showed that butyrate content was negatively correlated with OGTT, HOMA-IR of pharmacodynamic indexes, indicating the beneficial effect of butyrate in the development of T2D (Figures 3D, E).

### 3.4 Effect of QRZSF on colonic histomorphological changes

**FIGURE 4:**
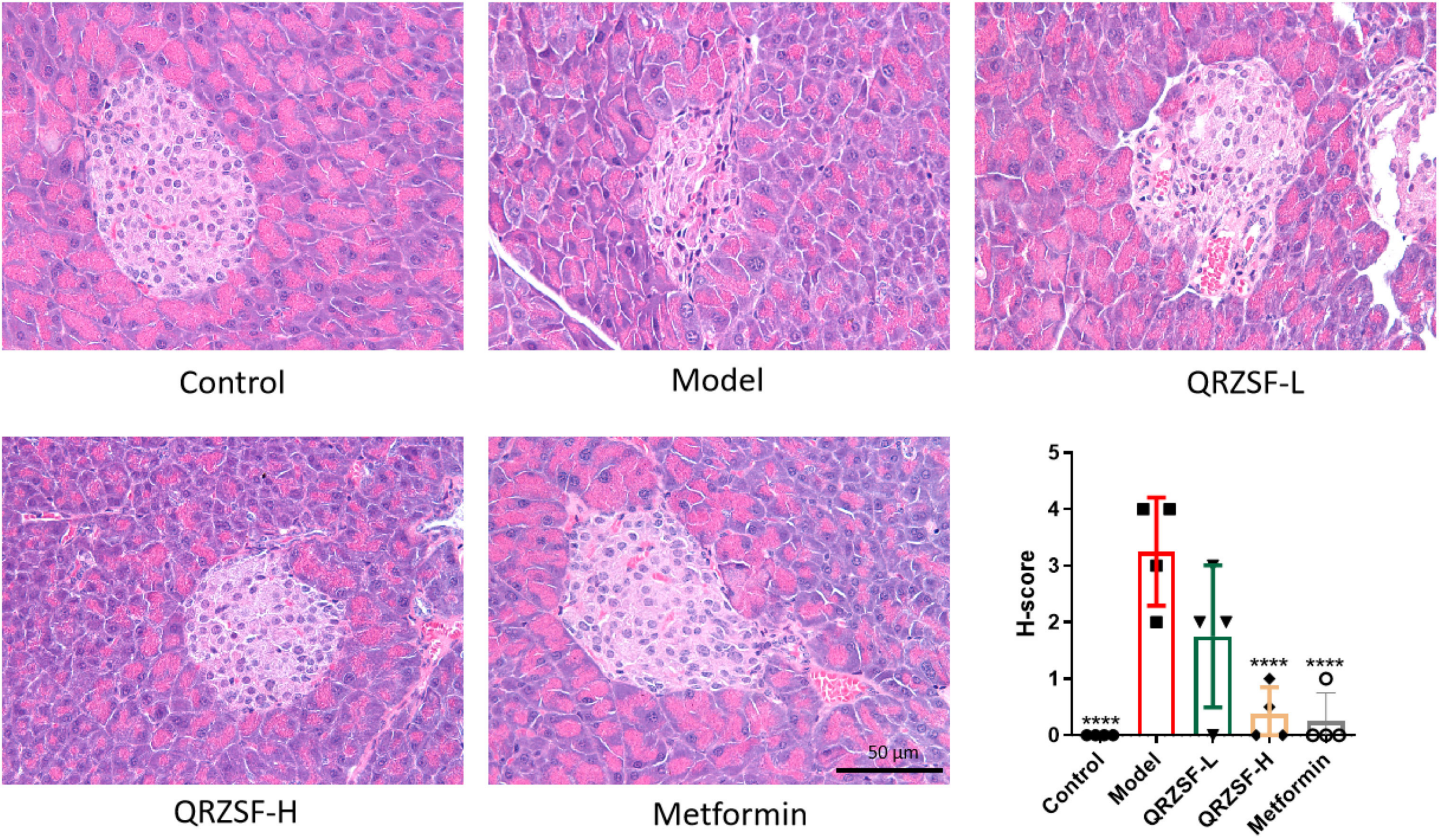
Changes of pathological sections of pancreatic tissue and score of histopathology injury degree after administration of (QRZSF). ****P < 0.0001.

Hematoxylin and eosin stains showed that the islets of the control group were circular in shape, with islet cells arranged in clusters and abundant capillaries between the cells (Figure 4). Pancreatic β cells were uniformed in size, with clear nuclear chromatin, clear cytoplasmic boundaries, and weakly staining. The size of the acini in the exocrine region was consistent and neatly arranged. The acinar cells contained many eosinophilic proenzyme particles, and no obvious degeneration or necrosis was observed. There was no congestion, edema, or inflammatory cell accumulation in the pancreatic stroma. The number of pancreatic islets in model group significantly decreased, the size of the islets decreased, the structure of the islets was damaged, the cell arrangement was disordered, the cells degenerated, and the cytoplasm was deeply stained. The arrangement of pancreatic β cells was sparse and uneven, and the morphology of cells was uneven. Some cells had vacuoles in their cytoplasm, which were unclear. No abnormalities were found in the acini of the exocrine department.

Metformin and QRZSF-H groups showed significant reduction in pancreatic islet lesions, and the cell morphology of the islets basically returned to normal compared with model group, while QRZSF-L group showed a slight reduction in pancreatic islet lesions. Histopathological scoring revealed significantly higher islet cell degeneration scores in the model group versus controls, while both metformin and QRZSF-H treatments markedly attenuated these degenerative changes, demonstrating significantly lower scores compared to the model group. It indicates that the damage in the model group was mainly in the pancreatic islets, manifested as structural disorder and partial cell degeneration and necrosis. Metformin and QRZSF-H group showed significant improvement in pathological changes.

### 3.5 Effect of QRZSF on gut microbiota

**FIGURE 5:**
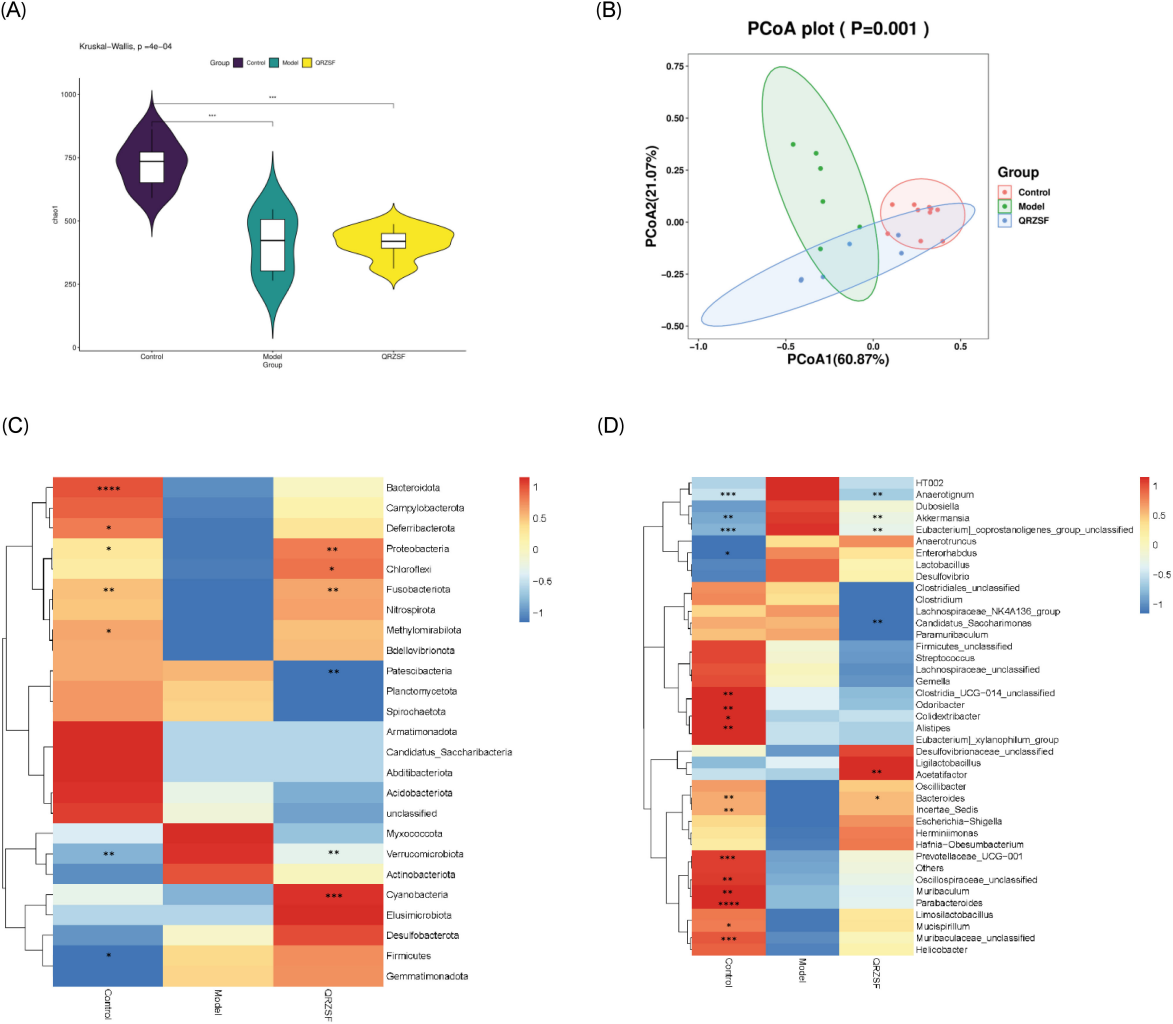
Gut microbiota sequencing by 16S rDNA in the type 2 diabetes (T2D) mouse after administration of Qing-Re-Zao-Shi-Jian-Pi prescription (QRZSF). **(A)** Alpha diversity analysis of gut microbiota; **(B)** Beta diversity analysis of gut microbiota; **(C)** Changes of phylum level; **(D)** Changes of genus level. *P < 0.05, **P < 0.01, ***P < 0.001, ****P < 0.0001.

We used Chao1 index to describe the community richness of intestinal microbiota. Significant decreases in Chao1 indices were observed in model and QRZSF-H groups versus controls. It was suggested that the model group and QRZSF-H group had a significant impact on the abundance of gut microbiota types in T2D mice (Figure 5A). Based on the principal co-ordinates analysis (PCoA) plot, it was shown that the distribution areas of the control group and model group were different, the structure of QRZSF-H group and control group was more similar (Figure 5B). Significant structural differences in gut microbiota composition were observed between control and model groups. The gut microbiota structure in the QRZSF-H group showed partial overlap with the model group but exhibited greater similarity to the control group.

We found that at the phylum level, the four dominant bacterial phyla of mice in the control group and the QRZSF-H group were *Firmicutes, Bacteroidota*, *Proteobacteria*, *Campylobacter*. The four dominant bacterial phyla of the model group mice were *Firmicutes*, *Bacteroidota*, *Proteobacteria*, *Verrucomicrobiota*. The Heatmap of species abundance clustering based on phylum level showed that the abundance of *Bacteroidota*, *Deferribacterota*, *Proteobacteria*, *Fusobacteriota*, and *Methylomirabilota* were decreased, while the abundance of *Verrucomicrobiota* and *Firmicutes* were increased in model group compared with the control group. The abundance of *Proteobacteria*, *Chloroflexi*, *Fusobacteriota*, and *Cyanobacteria* were increased, while the abundance of *Patescibacteria* and *Verrucomicrobiota* were decreased in QRZSF-H group compared with the model group.

Compared to the model group, the QRZSF-H group exhibited increased relative abundances of *Proteobacteria*, *Chloroflexi, Fusobacteriota*, and Cyanobacteria, alongside reduced abundances of *Patescibacteria* and *Verrucomicrobiota* (Figure 5C).

According to the genus level species abundance clustering Heatmap, the abundance of *Anaerotignum*, *Akkermansia*, Eubacterium_ Coprostanoligenes_ Group_ Unclassified and *Enterorhabdus* were increased, while *Clostridia_ UCG-014_ Unclassified*, *Odoribacter*, *Colidextribacter*, *Alistipes*, *Bacteroides*, *Incertae_ Sedis*, *Prevotellaceae_ UCG-001*, *Oscillospiraceae_unclassified*, *Muribaculum*, *Parabacteroides*, *Mucispirillum*, and *Muribaculaceae_ unclassified* showed significantly reduced abundance in the model group compared to controls. QRZSF-H group significantly altered gut microbiota composition compared to the model group, with decreased relative abundances of *Anaerotignum*, *Akkermansia*, *Eubacterium_ coprostanoligenes_ group_ unclassified*, *Candidatus_ Saccharimonas*, alongside increased abundances of *Acetatifactor* and *Bacteroides* (Figure 5D).

## 4 Discussion

Our research showed that the QRZSF can correct the disorder of glucose and lipid metabolism, reduce the level of proinflammatory factors, improve IR and pancreatic tissue damage, regulate the diversity of gut microbiota, and then effectively prevent and delay the occurrence and development of diabetes. This not only expands the clinical application scope of traditional famous prescriptions, but also aligns with the Traditional Chinese Medicine (TCM) principle of ’preventive treatment of disease, which is important for better excavating the treasure trove of traditional Chinese medicine and improving the quality of life and health level of mankind.

Diabetes is a group of chronic metabolic diseases with hyperglycemia as the common feature. The incidence of diabetes is growing rapidly worldwide, and more than 90% of them are T2D. Because of its complex metabolic disorder, it is easy to cause multi-system damage, and then cause serious complications. Timely prevention and proactive management play a crucial role in delaying the occurrence and development of diabetes. IR and abnormal insulin secretion are two important links in the pathogenesis of T2D, but there are individual differences in their relative contributions to the pathogenesis of this disease (9). Most of T2D patients have IR, which is manifested by the decreased sensitivity of target organs such as liver, skeletal muscle and fat to insulin and the inability to take up and use glucose normally. IR is a chronic inflammatory state. Body inflammatory factors can participate in and interfere with insulin signal transduction, affecting the normal role of insulin, leading to the occurrence of IR (10, 11). In the IR state, a large number of pro-inflammatory cytokines and inflammatory mediators are upregulated, especially TNF- α, Monocyte chemotactic protein-1 (MCP-1), C-reactive protein (CRP) and interleukin (IL) (12). TNF- α is one of the most important proinflammatory mediators, which can induce IR in adipocytes and surrounding tissues through suppression of lipoprotein esterase or reducing the activity of insulin receptor. IL-6 can promote the production and secretion of insulin in prediabetes, leading to the occurrence of hyperinsulinemia, which is associated with TNF- α. At the same time, it causes pancreatic islets β Cytotoxic effect of cells, leading to impaired glucose regulation and accelerated progression to diabetes (13). IL-1 β and islets β Cell function is negatively correlated, which can cause age-related islets β decreased cellular function (14). This study showed that blood glucose, insulin, HOMA-IR, TG, LDL-C, TCHO, IL-1β, TNF- α showed marked elevation in T2D mice, suggesting that there is hyperinsulinemia and IR in T2D, the body is in an inflammatory state, insulin can not be used normally, which is manifested as hyperglycemia. QRZSF-H can effectively reduce blood glucose, insulin, HOMA-IR and IL-1β, TNF- α in T2D mice. The results showed that QRZSF could inhibit the inflammatory reaction in T2D, improve glucose and lipid metabolism, and reduce IR.

At present, numerous studies have confirmed alterations in the composition of intestinal microbiota and its metabolites in T2D (15), and the dysregulation of intestinal microbial environment will damage the intestinal barrier. When the intestinal epithelial barrier function is weakened, intestinal microbes and their harmful metabolites can enter epithelial cells as antigens, prompting dendritic cells and macrophages to secrete proinflammatory cytokines while presenting antigens, further inducing low-grade systemic inflammation, affecting various metabolic organs, and finally exacerbating the process of IR. Therefore, the disruption of gut barrier integrity and the occurrence of chronic intestinal inflammation are the key factors for the pathogenesis of IR induced by intestinal microbiota (16, 17). This study observed that the gut microbiota of T2D mice demonstrated distinct variations compared with that of normal mice in terms of phylum level and genus level species abundance. *Verrucomicrobiota* was the unique dominant phylum of T2D model group. The gut microbiota of mice in the QRZSF-H group after the intervention is consistent with the dominant bacteria of normal mice, which are *Firmicutes, Bacteroidota, Proteobacteria, Campylobacter*. Compared with T2D model group, the abundance of *Verrucomicrobiota,Anaerotignum,Akkermansia, Eubacterium_coprostanoligenes_group_unclassified, Candidatus_Saccharimonas* decreased significantly, while the abundance of *Acetatifactor* and *Bacteroides* increased significantly in the QRZSF-H group.

Intestinal microbiota ferment undigested and unabsorbed dietary fiber, which generates SCFAs as metabolites. Numerous studies have demonstrated a significant association between SCFAs and T2D (18, 19). SCFAs can regulate host metabolism by maintaining energy balance, regulating glucose and lipid metabolism, maintaining the intestinal barrier, and reducing inflammatory reactions (20). Acetate is produced by *Bacteroides, Bifidobacterium, Eubacterium, Ruminococcus, Peptostreptococcus, Clostridium*, and *Streptococcus species.* Propionate is produced by *Clostridium species.* Butyrate is produced by *Bacteroides, Eubacterium*, and *Clostridium species* (21). Acetate and butyrate have been found to significantly increase glucagon-like peptide (GLP-1) secretion in the colon and, to a lesser extent, peptide Tyrosin-Tysrosin (PYY) secretion, which in turn exerts hypoglycemic effects (22). Butyrate not only improves pancreatic islets β Cell function, but also by improving intestinal barrier function and inhibiting NF- κ B signaling, which exerts direct anti-inflammatory activity and reduces local and systemic inflammatory responses (23). This study found that the contents of butyrate and propionate in intestinal SCFAs of T2D mice decreased significantly, while the content of acetate did not change significantly compared with normal mice. After the intervention of QRZSF-H, the butyrate content of T2D mice increased significantly, which could be linked to the change of gut microbiota diversity in mice. It has been shown that T2D is associated with a decrease in butyrate-producing genera (24), then increasing butyrate-producing genera may have a favorable impact on T2D therapy. This is consistent with our findings. From the intestinal microbiota perspective, this study demonstrated a substantial rise in *Bacteroides* abundance in the QRZSF-H intervention group. *Bacteroides* are known to be the main genus producing butyrate, indicating that *Bacteroides* may significantly contribute to the treatment of T2D by affecting SCFAs, but the specific mechanism of action remains unclear.

Emerging evidence indicates that gut microbiota significantly contributes to the occurrence and development of T2D through the metabolic pathway of SCFA (25). Among individuals with diabetes, alterations in intestinal microbiota composition and SCFA metabolite levels influence inflammatory status, resulting in IR (26).

## 5 Conclusion

Our study verified that QRZSF-H can effectively reduce blood glucose, blood lipids, IR, inflammatory response and pancreatic damage in type 2 diabetic mice. Meanwhile, QRZSF-H can modulate gut microbiota diversity and increase butyrate levels. However, this study has limitations. The key intestinal flora affecting IR of T2D is still unclear, and the underlying mechanisms remain to be elucidated.

## Data Availability

The raw data supporting the conclusions of this article will be made available by the authors, without undue reservation.
