# Correction: Gut microbiota and targeted metabolomics of short-chain fatty acid reveals Qing-Re-Zao-Shi-Jian-Pi prescription on glucose and lipid metabolism in type 2 diabetic mice

**DOI:** 10.3389/fnut.2025.1750874

**Published:** 2026-01-09

**Authors:** Jing Liu, Meng Qin, Haidan Wang, Yingzhuo Ran, Xin Shao

**Affiliations:** 1 Department of Endocrinology, Nanjing Hospital of Chinese Medicine Affiliated to Nanjing University of Chinese Medicine, Nanjing, Jiangsu, China; 2 State Key Laboratory of Natural Medicines, School of Traditional Chinese Pharmacy, China Pharmaceutical University, Nanjing, Jiangsu, China; 3 Jiangsu Province Hospital of Chinese Medicine, Affiliated Hospital of Nanjing University of Chinese Medicine, Nanjing, Jiangsu, China

**Keywords:** type 2 diabetes, microbiota, short-chain fatty acid, Chinese medicine, insulin resistance

In the **Abstract**, the words “*Chioroflexi*” and “*Acetofactor*” were misspelled. These words have been corrected to “*Chloroflexi*” and “*Acetatifactor*.”

In the **Background**, paragraph three, the words “*Chioroflexi*,” “*Patecibacteria*,” “*Candida_ Saccharimonas*,” and “*Acetofactor*” were misspelled. These words have been corrected to “*Chloroflexi*,” “*Patescibacteria*,” “*Candidatus_ Saccharimonas*,” and “*Acetatifactor*.”

In the **Results**, *Effect of QRZSF on gut microbiota*, paragraph two, the words “*Chioroflexi*” and “*Patecibacteria*” were misspelled. These words have been corrected to “*Chloroflexi*” and “*Patescibacteria*.”

In the **Results**, *Effect of QRZSF on gut microbiota*, paragraph four, the words “*Enterohabdus*,” “*naerotignum*,” “*Candida_ Saccharimonas*,” and “*Acetofactor*” were misspelled. These words have been corrected to “*Enterorhabdus,” “Anaerotignum*,” “*Candidatus_ Saccharimonas*,” and “*Acetatifactor*.”

In the **Discussion**, paragraph three, the words “*Candida_ Saccharimonas*” and “*Acetofactor*” were misspelled. These words have been corrected to “*Candidatus_ Saccharimonas*,” and “*Acetatifactor*.”

The original version of this article has been updated.

